# Oxymatrine Downregulates *HPV16E7* Expression and Inhibits Cell Proliferation in Laryngeal Squamous Cell Carcinoma Hep-2 Cells In Vitro

**DOI:** 10.1155/2015/150390

**Published:** 2015-02-24

**Authors:** Xin-Jiang Ying, Bin Jin, Xin-Wei Chen, Jin Xie, Hong-Ming Xu, Pin Dong

**Affiliations:** Department of Otolaryngology-Head and Neck Surgery, Shanghai First People's Hospital, Shanghai Jiao Tong University, Shanghai 200080, China

## Abstract

*Objective*. To investigate the possible mechanisms of oxymatrine's role in anti laryngeal squamous cell carcinoma. *Methods*. We examined the effects of oxymatrine on the proliferation, cell cycle phase distribution, apoptosis, and the protein and mRNA expression levels of *HPV16E7* gene in laryngeal carcinoma Hep-2 cells in vitro. The *HPV16E7* siRNA inhibition was also done to confirm the effect of downregulating *HPV16E7* on the proliferation in Hep-2 cells. *Results*. Oxymatrine significantly inhibited the growth and proliferation of Hep-2 cells in a dose-dependence and time-dependence manner. Oxymatrine blocked Hep-2 cells in G0/G1 phase, resulting in an obvious accumulation of G0/G1 phase cells while decreasing S phase cells. Oxymatrine induced apoptosis of Hep-2 cells, whose apoptotic rate amounted to about 42% after treatment with 7 mg/mL oxymatrine for 72 h. Oxymatrine also downregulated the expression of *HPV16E7* gene, as determined by the western blotting and reverse transcription-polymerase chain reaction analysis. Knockdown of *HPV16E7* effectively inhibited the proliferation of Hep-2 cells. *Conclusions*. Oxymatrine inhibits the proliferation and induces apoptosis of laryngeal carcinoma Hep-2 cells, which might be mediated by a significant cell cycle arrest in G0/G1 phase and downregulation of *HPV16E7* gene. Oxymatrine is considered to be a likely preventive and curative candidate for laryngeal cancer.

## 1. Introduction

Laryngeal squamous cell carcinoma is a common malignancy in the head and neck region [1]. In addition to the well-established risk factors for laryngeal cancer such as tobacco smoking and alcohol consumption, a number of studies suggest that human papillomavirus type 16* (HPV16)* is also associated with the development of laryngeal cancer [2–4]. Carcinogenesis by* HPV16 *is primarily attributed to the continuous expression of viral protein* E6/E7*, which results in cellular resistance to apoptosis and cancer formation by, respectively, interacting with* p53* and* pRb* in cell cycle checkpoint [5–7]. We have also demonstrated that the overexpression of* HPV16E7* oncoprotein is associated with the development of laryngeal cancer [8]. Therefore, the* HPV16E7* oncogene is considered to be potential therapeutic target for blocking the development of laryngeal cancer.

Treatment strategies for laryngeal cancer today are focused on larynx preservation which have the aim to preserve not only the anatomic organ, but, more importantly, also its function [9]. To achieve organ preservation, various options for treatment modalities including radiotherapy, chemotherapy, and targeted molecular therapies have been added to the conventional approaches of surgery [10]. Although a number of chemotherapeutic drugs are available for the treatment of cancer, which can be used for controlling the growth of cancer and have received certain curative efficacy, the side effects limit their application. Therefore, to discover novel natural substances that have therapeutic selectivity without significant toxicity to normal cells is an important tendency for laryngeal cancer therapy. Oxymatrine is one of the quinolizidine alkaloids extracted from the root of traditional Chinese herbal medicine* Sophora japonica (Sophora flavescens *Ait*).* It has been reported that oxymatrine plays important roles in anti-inflammation, inhibition of immune reaction, antivirus, antitumors, and so on [11–14]. Different from the usual chemotherapy medicine, oxymatrine has the selectivity kill capability to the tumor cells, with little influence to some normal cells [15].

To our knowledge, there are few studies on the application of oxymatrine in the treatment of laryngeal cancer. Similarly, there currently is no report concerning the mechanism of oxymatrine and its putative relationship with* HPV16E7*. In the present study, we investigated the effects of oxymatrine on laryngeal squamous cell carcinoma Hep-2 cells by examining cell proliferation, cell cycle phase distribution, apoptosis, and the protein and mRNA expression levels of* HPV16E7* gene in vitro. This study aimed to explore the antitumor mechanisms of oxymatrine and provide experimental evidence for the application of oxymatrine in the prevention and treatment of laryngeal squamous cell carcinoma.

## 2. Materials and Methods

### 2.1. Oxymatrine

Oxymatrine (300 mg/mL) was purchased from Chia Tai Tianqing Pharmaceutical Group Co., Ltd., Nanjing (Jiangsu, China). In the experiment, we used the same batch of oxymatrine, whose purity is more than 99% indicated by SDS-PAGE analysis.

### 2.2. Cell Line and Culture

The Hep-2 human laryngeal carcinoma cell line was obtained from the American Type Culture Collection (ATCC, Manassas, VA, USA). Cells were routinely cultured in Dulbecco's modified Eagle's medium (DMEM; Gibco Corporation, Carlsbad, CA, USA) supplemented with 10% fetal bovine serum (FBS; Sijiqing, China), 100 U/mL penicillin G, and 100 U/mL streptomycin (Gibco, Carlsbad, CA, USA) in a humidified atmosphere of 95% air and 5% CO_2_ at 37°C. The medium was changed every 3 days.

### 2.3. Proliferation Assay

Hep-2 cells in logarithmic growth phase were seeded in 96-well microplates with 1 × 10^5^ each well and cultured cells with DMEM growth medium for 24 h. Then the medium was replaced with DMEM growth medium containing various concentrations of oxymatrine (3, 5, and 7 mg/mL) and cultured continuously. In addition, control cells were incubated with medium only. After exposure to oxymatrine, changes of cell morphology were observed by optical microscope (Olympus, Japan) and the proliferation of Hep-2 cells was assessed by using CCK-8 assay. After 24, 72, 120, and 168 h, cells were treated with 10 *μ*L of CCK-8 reagent (Dojindo Molecular Technologies, Kunamoto, Japan) and incubated at 37°C for 1 h. An automatic microtiter plate reader was set to zero according to the control wells. The absorbance (*A*) of each well was measured at a wavelength of 450 nm. The rate of inhibition was calculated as follows: cell proliferation inhibition (%) = (1 − average absorbance (*A*) of the experimental group/average absorbance (*A*) of the control group) × 100%.

### 2.4. Cell Cycle Analysis

After preincubation with DMEM growth medium for 24 h, Hep-2 cells were exposed to medium containing different concentrations of oxymatrine (0, 3, 5, and 7 mg/mL) for 72 h. The cells were harvested by centrifugation, fixed in 70% cooled ethanol, and then dyed with propidium iodide (PI) for 30 min. DNA content was detected by a flow cytometer (FACSCaliburTM, America BD). The relative proportions of cells in the individual cell-cycle phase fraction were determined from the flow cytometry data.

### 2.5. Apoptosis Assay

All the operations were based on instructions which are described as manual of cell apoptosis assay kit. After treatment with various concentrations of oxymatrine (0, 3, 5, and 7 mg/mL) for 72 h, Hep-2 cells were harvested by centrifugation, resuspended in binding buffer, and successively incubated with 5 *μ*L of Annexin V-FITC and 5 *μ*L of PI (Multi Sciences, China) for 15 min at room temperature. Apoptosis was determined by flow cytometric analysis using a flow cytometer (FACSCaliburTM, America BD).

### 2.6. Real-Time PCR

Total RNA was extracted from Hep-2 cells treated with 5 mg/mL of oxymatrine for 72 h or the control cells using Trizol reagent (Invitrogen, Shanghai, China) and converted to cDNA using MMLV reverse transcriptase kit (Promega, USA). Aliquots of cDNA were subjected to quantitative real-time PCR using Step One Plus Real-time PCR system (Applied Biosystems, USA). The mRNA levels were normalized to that of *β-actin*. The specific primer pairs were as follows:* HPV16E7*, sense: 5′-CGGGATCCATGCATGGAGATACA-3′ and antisense: 5′-GCG­GGC­CCT­TAT­GGT­TTC­TGA­GA-3′; *β-actin*, sense: 5′-CAT­GTA­CGT­TGC­TAT­CCA­GGC-3′ and antisense: 5′-CTC­CTT­AAT­GTC­ACG­CAC­GAT-3′. Data were analyzed using the 2^−ΔΔCt^ method.

### 2.7. Western Blot Analysis

The protein was extracted from Hep-2 cells treated with 5 mg/mL of oxymatrine for 72 h or the control cells. Cell extracts were prepared in radioimmunoprecipitation assay buffer (150 mM NaCl, 0.1% SDS, 0.5% sodium deoxycholate, 1% Nonidet P-40, and 50 mM Tris, pH 8.0), with the addition of 2 mM phenylmethylsulfonyl fluoride. Lysis buffer was freshly prepared and added in 6-well plates (100 *μ*L/well) on ice, which were then incubated for 10 min. Protein concentrations were determined by protein assay kit. Equal amounts of proteins (10 *μ*g per condition) were boiled for 10 min in loading buffer before being separated on 15% SDS-PAGE gels. Separated proteins were transferred to polyvinylidene difluoride membranes at 100 V for 1 h before membrane blocking in PBS with 5% skim milk powder and Tween 20. Anti-*HPV16E7* primary antibody (Biorbyt) and secondary antibodies (Abcam) were diluted by 1/500 with PBST buffer and incubated for 60 min at room temperature. Membrane was washed for 3 times by PBST before each step. Protein bands were visualized by enhanced chemiluminescence. *β-actin* was used as an internal control.

### 2.8. RNA Interference

The RNAi sequence for* HPV16E7* (GCT TCG GTT GTG CGT ACA A) was identified by using the manufacturer's RNAi Designer programme, and the negative control having no homology with human genome was created by a scrambled sequence (TTC TCC GAA CGT GTC ACG T). The siRNA duplex was transfected using Lipofectamine 2000 Reagent (Invitrogen) as recommended by the manufacturer, and the cells were assayed for silencing 72 h after transfection.

### 2.9. Statistical Analysis

All experiments were performed in triplicate, and data were shown as the mean ± SD where they are applicable. Statistically significant differences between groups were determined by one-way ANOVA using SPSS 17.0 software (SPSS, Chicago, IL, USA), and *P* < 0.05 was considered statistically significant.

## 3. Results

### 3.1. Oxymatrine Inhibits the Growth and Proliferation of Hep-2 Cells

**Figure 1:**
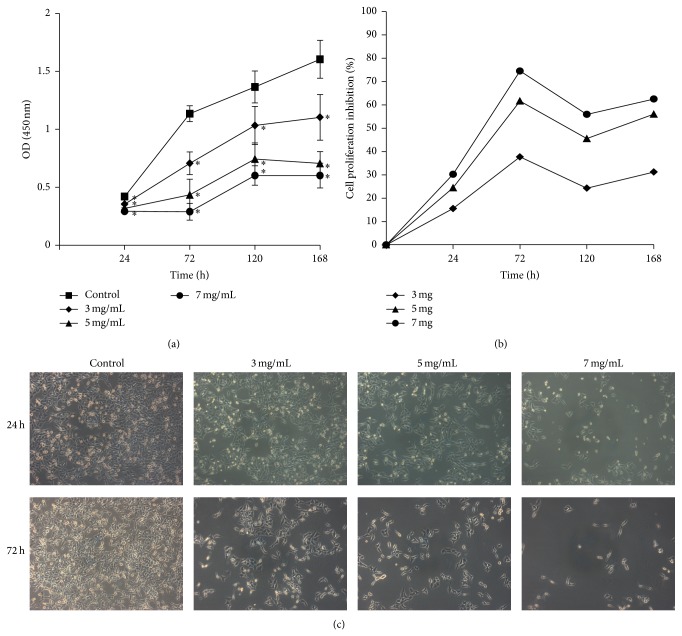
Effects of oxymatrine on proliferation of Hep-2 cells. (a) Hep-2 cells were treated with oxymatrine at different concentrations for various times and the OD values were obtained through reading plate at 450 nm with 96-well microtest spectrophotometer by CCK-8 assay. (b) Relationship between the percentage of laryngeal cancer cell proliferation inhibition and different concentrations of oxymatrine. (c) Morphological changes induced by various concentrations of oxymatrine for 24 and 72 h in Hep-2 cells (×100).

To determine whether oxymatrine inhibits the proliferation of Hep-2 cells, we examined the effect of oxymatrine on proliferation of the Hep-2 cell line by using CCK-8 assay. We found that oxymatrine significantly inhibited the growth of Hep-2 cells in a concentration-dependence and time-dependence manner, compared with that in the control cells (*P* < 0.05, Figure 1(a)). In the group treated with 3 mg/mL of oxymatrine at the indicated time points of 24, 72, 120, and 168 h, the percentage of Hep-2 cell proliferation inhibition was 15.6%, 37.7%, 24.3%, and 31.2%, respectively. Additionally, in the group treated with 5 mg/mL of oxymatrine, the percentage of cell proliferation inhibition at 24, 72, 120, and 168 h was 24.5%, 61.6%, 45.5%, and 56.0%, respectively. In the group treated with 7 mg/mL of oxymatrine, the percentage of cell proliferation inhibition at 24, 72, 120, and 168 h was 30.2%, 74.5%, 55.9%, and 62.5%, respectively (Figure 1(b)). At 72 h, oxymatrine showed a significantly higher inhibiting effect than that at 24 h. In contrast, there was no significant difference in cell proliferation inhibition among prolonged treatment for 120 h and 168 h. Therefore, we choose the time point of 72 h for the further investigation. Besides, we also found that the number of cells decreased with increasing concentrations of oxymatrine under a microscope (Figure 1(c)).

### 3.2. Oxymatrine Arrests Hep-2 Cells in the G0/G1 Phase

**Figure 2:**
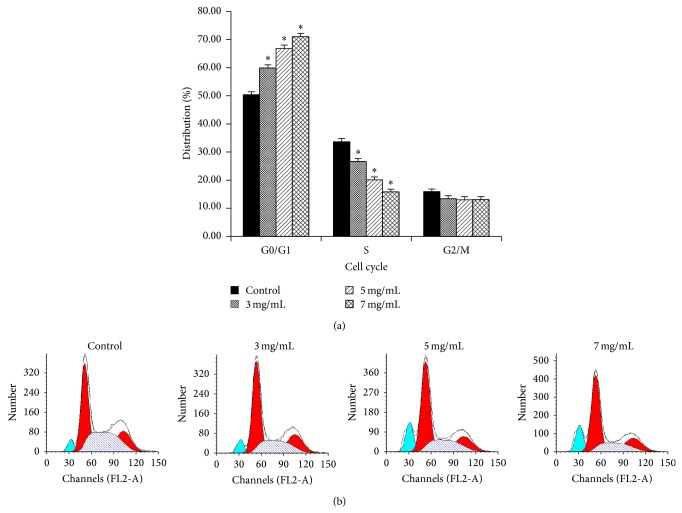
Changes of cell cycle distribution in Hep-2 cells after treatment with oxymatrine at different concentrations for 72 h. (a) Oxymatrine increased the proportion of cells in G0/G1 phase and decreased that in the S phase, compared with the control group (^*^ *P* < 0.05). (b) FCM analysis showed oxymatrine arrested Hep-2 cell cycling in the G0/G1 phase.

To evaluate the effect of oxymatrine on the cell cycle distribution, the FCM analysis was performed. We found that the proportion of G0/G1 phase was 59.91 ± 1.09%, 66.85 ± 1.18%, and 71.07 ± 1.12%, respectively, after treatment with 3, 5, and 7 mg/mL of oxymatrine for 72 h. For the control group, the percentage of G0/G1 phase was 50.37 ± 1.08%. The frequency of Hep-2 cell in G0/G1 phase is significantly increased while that of cells in the S-phase is decreased with the increasing concentration of oxymatrine, as compared with that in the control group (*P* < 0.05, *n* = 6, Figure 2). These data indicated that oxymatrine arrested cell cycle of Hep-2 cells in G0/G1 phase.

### 3.3. Oxymatrine Induces Apoptosis of Hep-2 Cells

**Figure 3:**
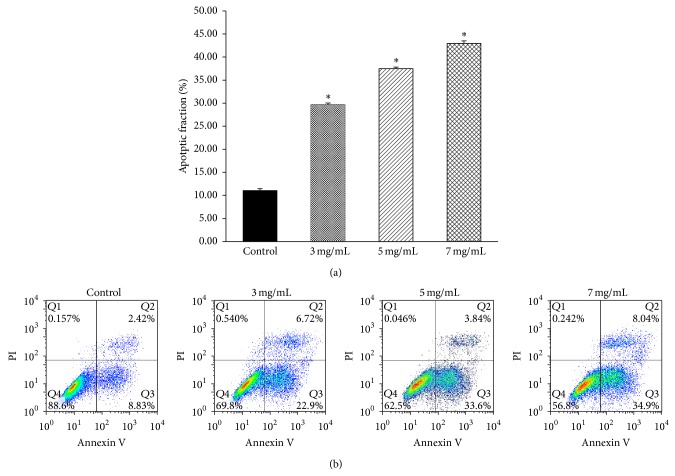
Apoptosis induced by oxymatrine was detected by Annexin V-FITC/PI. (a) Oxymatrine significantly induced cell apoptosis in the treatment group, compared with that in the control group (^*^ *P* < 0.05). (b) FCM data showed that the apoptotic rate of Hep-2 cells treated with oxymatrine was significantly increased.

To further investigate the effects of oxymatrine on cell death of Hep-2 cells, the rate of apoptosis was evaluated by flow cytometry analysis. We found that the apoptosis rate was 29.62 ± 0.41%, 37.44 ± 0.38%, and 42.94 ± 0.56%, respectively, after treatment with 3, 5, and 7 mg/mL of oxymatrine for 72 h. For the control group, the apoptosis rate was 11.05 ± 0.40%. The percentage of apoptosis is significantly increased with the increasing concentration of oxymatrine, as compared with that in the control group (*P* < 0.05, *n* = 6, Figure 3). The results demonstrate that suppression of cell proliferation may be caused by cell death resulting from oxymatrine.

### 3.4. Oxymatrine Downregulates* HPV16E7* Expression in Hep-2 Cells

**Figure 4:**
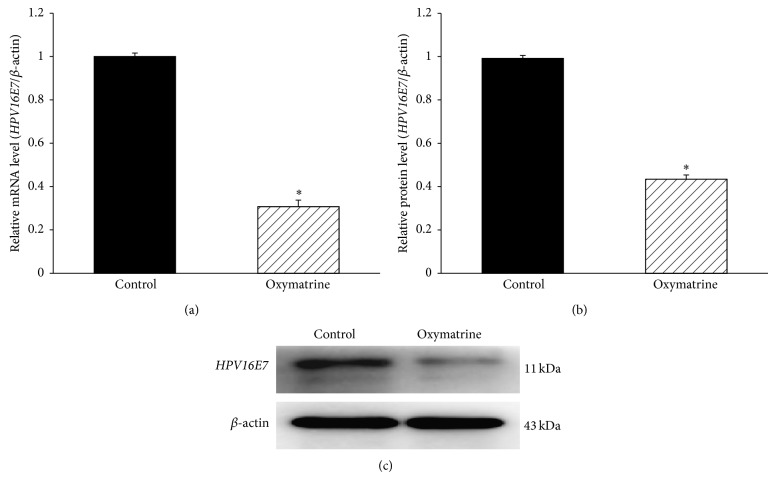
Expressions of* HPV16E7* mRNA and protein in Hep-2 cells treated with oxymatrine. (a) The expression levels of* HPV16E7* mRNA were assessed by qRT-PCR (2^−ΔΔCt^ method. 2^−ΔΔCt^ indicated relative expression level in the treatment group, compared with that in the control group). (b) The protein level of* HPV16E7* in the treatment group was significantly reduced, compared with the control group (^*^ *P* < 0.05). (c) The expression levels of* HPV16E7* protein were detected by Western blotting.

The effect of oxymatrine on the expression of* HPV16E7* gene was examined by real-time PCR and Western blot analysis. We found that both mRNA and protein levels of* HPV16E7* gene in the Hep-2 cells treated with 5 mg/mL of oxymatrine were sharply reduced, compared with that in the control group (*P* < 0.05, *n* = 6, Figure 4). The mRNA level is decreased by 69.3%, while the protein level is reduced by 56.3%. These data demonstrate that the expression of* HPV16E7* gene is efficiently downregulated by oxymatrine in Hep-2 cells.

### 3.5. Knockdown of* HPV16E7* Inhibits the Proliferation of Hep-2 Cells

**Figure 5:**
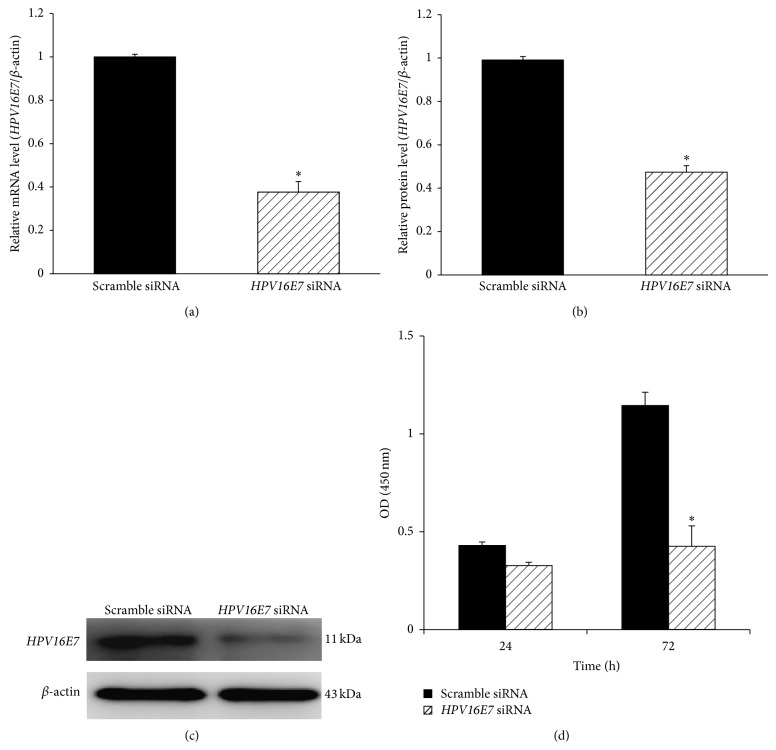
Expressions of* HPV16E7* mRNA and protein in Hep-2 cells treated with* HPV16E7* siRNA. (a) The expression levels of* HPV16E7* mRNA were assessed by qRT-PCR (2^−ΔΔCt^ method. 2^−ΔΔCt^ indicated relative expression level in the treatment group, compared with that in the control group). (b) The protein level of* HPV16E7* in the* HPV16E7* siRNA group was significantly reduced, compared with the scramble control group (^*^ *P* < 0.05). (c) The expression levels of* HPV16E7* protein were detected by Western blotting. (d) Hep-2 cells were treated with* HPV16E7* siRNA and proliferation was measured by CCK-8 assay at 24 and 72 h after treatment.

To assess the effect of downregulating* HPV16E7* on the proliferation of Hep-2 cells, we knocked down* HPV16E7* mRNA transcripts using* HPV16E7* siRNA.* HPV16E7* siRNA caused over 62.4% of reduction in* HPV16E7* mRNA expression and 52.7% in protein expression compared to scrambled siRNA (Figures 5(a)–5(c)). After 72 h of culture, the proliferation inhibition of Hep-2 cells transfected by* HPV16E7* siRNA was 62.8% compared with scramble control (*P* < 0.05, Figure 5(d)). These data indicated that downregulating* HPV16E7* inhibited the growth of Hep-2 cells.

## 4. Discussion

Oxymatrine is one of the main alkaloid components in the traditional Chinese herbal medicine* Sophora japonica (Sophora flavescens *Ait). It is commonly known that oxymatrine exhibits many biological activities and possesses a wide range of pharmacological effects, such as anti-inflammation [16], antifibrosis [17], analgesic [18], immunosuppression [19], and antivirus [20]. Oxymatrine also has been extensively studied, for their cancer chemoprevention potentially against various cancers, for instance, gastric cancer [21], breast cancer [22], hepatocellular carcinoma [23], and pancreatic cancer [24]. However, the mechanisms of the antitumor properties of oxymatrine in laryngeal cancer are not well established to date. In the present study, we treated laryngeal squamous cell carcinoma Hep-2 cells with various concentrations of oxymatrine, to investigate the effect of oxymatrine on the cell proliferation, cell-cycle progression, and apoptosis of laryngeal cancer.

The loss of regulatory control of cell proliferation and apoptosis plays an important role in tumorigenesis. It has been a well-studied area to interfere with the dysfunctions of cell proliferation and apoptosis in cancer research. In this study, we found that oxymatrine significantly inhibited the growth and proliferation in Hep-2 cells, which presented a concentration-dependence and time-dependence manner, as determined by the CCK-8 assay. Not only does the inhibitory mechanism involve alteration of the proliferation of cancer cells, but also induction of apoptosis. From apoptosis assay, we found that oxymatrine induced an obviously apoptosis in Hep-2 cells, and the apoptotic rate amounted to 42.94 ± 0.56% after being treated by 7 mg/mL oxymatrine for 72 h. In general, there is a near correlativity between apoptosis and cell cycle. If cell cycle is blocked in a phase, apoptosis would be appearing in the phase in which cell cycle is arrested. As shown from FCM analysis, oxymatrine significantly blocked the cell cycle of Hep-2 cells in G0/G1 phase, the cells in G0/G1 phase were significantly increased, while the cells in the S phase were decreased with the increasing concentration of oxymatrine, which suggests that apoptosis of Hep-2 cells induced by oxymatrine maybe occurs in G0/G1 phase. One of the basic characteristics of tumor cells is uncontrolled cell growth. The mechanism underlying uncontrolled proliferation is the destruction of the cell cycle control machinery. Regulation of cell cycle progression can potentially trigger cellular apoptosis suppressing cell proliferation to achieve antitumor effects. The results of our study demonstrate that oxymatrine may be a potential chemotherapeutics for the treatment of laryngeal cancer.

Molecular epidemiologic evidences clearly indicate that* HPV16* is closely associated with the development of laryngeal cancer, which encodes the* E6* and* E7* oncogenes, whose expression is essential for virus replication. The viral protein* E7* of* HPV16* binds to hypophosphorylated members of the retinoblastoma family, resulting in their destabilization and the disruption of* Rb/E2F* complexes [25], which repress transcription of genes required for cell cycle progression to allow the progression of cell cycle into S phase [26] and increase antiapoptotic protein* Bcl-2* to promote the survival of cells and stimulate cell proliferation [27]. In previous studies, oxymatrine has been demonstrated to exhibit specific pharmacological properties for antihepatitis virus. Although there are no reports on anti-*HPV* effects of oxymatrine, we found that oxymatrine downregulated the* HPV16E7* expression at both mRNA and protein levels. To assess the effect of downregulating* HPV16E7* on the proliferation of Hep-2 cells, we also knocked down* HPV16E7* mRNA transcripts using* HPV16E7* siRNA and found that downregulating* HPV16E7* inhibited the growth of Hep-2 cells. Thus, we speculate that oxymatrine may inhibit proliferation of Hep-2 cells through downregulation of* HPV16E7* gene. In other studies, compounds isolated from arsenic have demonstrated inhibitory effects on* HPV *oncogene activation and anti-*HPV*-associated tumors [28]. Therefore, we hypothesize that oxymatrine may also have an anti-*HPV* effect that may be able to treat laryngeal cancer. Moreover, oxymatrine has been found to enhance the antitumor immune response, which may also be beneficial for the treatment of laryngeal cancer.

In summary, our in vitro experiments demonstrate that oxymatrine significantly inhibits growth and proliferation and induces apoptosis of laryngeal carcinoma Hep-2 cells. This apoptosis may be in part mediated by a significant blockage in G0/G1 phase. We suggest that the observed effects of oxymatrine are likely associated with the regulation of the* HPV16E7* and downstream protein expression. Due to the effective, nontoxic, and natural antitumor properties, oxymatrine is considered to be a likely preventive and curative candidate for laryngeal cancer. Additional studies are required to determine the underlying mechanisms whereby oxymatrine suppresses laryngeal cancer at the molecular level and to provide a theoretical basis for the tumoricidal and clinical utility of oxymatrine.
